# Insulin/IGF-1 Signaling Is Downregulated in Barrett’s Esophagus Patients Undergoing a Moderate Calorie and Protein Restriction Program: A Randomized 2-Year Trial

**DOI:** 10.3390/nu13103638

**Published:** 2021-10-17

**Authors:** Diletta Arcidiacono, Alice Zaramella, Federico Fabris, Ricardo Sánchez-Rodríguez, Daniele Nucci, Matteo Fassan, Mariateresa Nardi, Clara Benna, Chiara Cristofori, Tiziana Morbin, Salvatore Pucciarelli, Alberto Fantin, Stefano Realdon

**Affiliations:** 1 Gastroenterology Unit, Veneto Institute of Oncology IOV-IRCCS, Via Gattamelata 64, 35128 Padua, Italy; alice.zaramella@iov.veneto.it (A.Z.); chiara.cristofori@iov.veneto.it (C.C.); tiziana.morbin@iov.veneto.it (T.M.); alberto.fantin@iov.veneto.it (A.F.); stefano.realdon@iov.veneto.it (S.R.); 2 Department of Surgery, Oncology and Gastroenterology (DiSCOG), University of Padua, Via Giustiniani 2, 35128 Padua, Italy; 3 Department of Biomedical Sciences, University of Padua, Viale Colombo 3, 35121 Padua, Italy; federico.fabris.5@phd.unipd.it (F.F.); ricardo.sanchezrodriguez@unipd.it (R.S.-R.); 4 Fondazione Istituto di Ricerca Pediatrica, Città della Speranza, Corso Stati Uniti 4, 35127 Padua, Italy; 5 Nutritional Support Unit, Veneto Institute of Oncology IOV-IRCCS, Via Gattamelata 64, 35128 Padua, Italy; daniele.nucci@iov.veneto.it (D.N.); mariateresa.nardi@iov.veneto.it (M.N.); 6 Department of Medicine (DIMED), Surgical Pathology Unit, University of Padua, Via Gabelli 61, 35121 Padua, Italy; matteo.fassan@unipd.it; 7 Veneto Institute of Oncology IOV-IRCCS, Via Gattamelata 64, 35128 Padua, Italy; 8 First Surgical Clinic, Department of Surgery, Oncology and Gastroenterology (DiSCOG), University of Padua, Via Giustiniani 2, 35128 Padua, Italy; clara.benna@unipd.it (C.B.); puc@unipd.it (S.P.)

**Keywords:** calorie–protein restriction, Barrett’s esophagus, insulin/IGF-1 signal transduction, esophageal adenocarcinoma prevention, obesity

## Abstract

Obesity and associated insulin resistance (Ins-R) have been identified as important risk factors for esophageal adenocarcinoma development. Elevated calories and protein consumption are also associated with Ins-R and glucose intolerance. We investigated the effect of a 24-month moderate calorie and protein restriction program on overweight or obese patients affected by Barrett’s esophagus (BE), as no similar dietary approach has been attempted to date in this disease context. Anthropometric parameters, levels of serum analytes related to obesity and Ins-R, and the esophageal insulin/IGF-1 signaling pathway were analyzed. This study is registered with ClinicalTrials.gov, number NCT03813381. Insulin, C-peptide, IGF-1, IGF-binding protein 3 (IGFBP3), adipokines, and esophageal expression of the main proteins involved in insulin/IGF-1 signal transduction were quantified using Luminex-XMAP^®^ technology in 46 patients who followed the restriction program (IA) and in 54 controls (CA). Body mass index and waist circumference significantly decreased in 76.1% of IA and 35.2% of CA. IGF-1 levels were reduced in 71.7% of IA and 51.8% of CA. The simultaneous reduction of glycaemia, IGF-1, the IGF-1/IGFBP3 ratio, and the improvement in weight loss-dependent insulin sensitivity, were associated with the downregulation of the insulin/IGF-1 signal on BE tissue. The proposed intervention program was an effective approach to counteract obesity-associated cancer risk factors. The improvement in metabolic condition resulted in a downregulation of the ERK-mediated mitogenic signal in 43.5% of patients, probably affecting the molecular mechanism driving adenocarcinoma development in BE lesions.

## 1. Introduction

Despite the general improvement in Western countries’ living conditions in recent decades, the incidence and prevalence of chronic diseases are increasing [1]. Obesity, central adiposity, type 2 diabetes, and insulin resistance are widespread. Their role as risk factors for oncological diseases is well documented [2,3,4], including in the context of esophageal adenocarcinoma (EAC) and its precancerous lesion, Barrett’s esophagus (BE) [5,6,7]. Barrett’s esophagus, a metaplastic change resulting from long-standing reflux disease, is identified in 5-15% of chronic reflux-affected patients [8]. Although the rate of EAC development in BE patients is low, its mortality rate is higher than 80% after 5 years from diagnosis [9,10], suggesting that it is important to improve early cancer diagnosis and prevention. BE patients are usually enrolled in expensive endoscopic follow-up programs for early cancer diagnosis. These programs are based on endoscopic biopsies standard protocols (four-quadrant biopsies taken at the gastroesophageal junction and 2 cm above it), but the effectiveness of this approach is unclear [11,12,13]. Thus, other factors and strategies need to be considered to reduce EAC risk.

In 2007, the World Cancer Research Fund (WCRF) and the American Institute for Cancer Research (AICR) assessed the link between diet, nutrition, physical activity, and cancer [14] and summarized their findings into 10 recommendations to be followed to prevent cancer. In a large prospective study, Romaguera et al. have demonstrated the concordance between adherence to such indications and the risk of the onset of various types of cancer (including cancer of the esophagus) [15].

The excessive amount of weight in obese patients is correlated with insulin resistance in different tissues, compensated for by the stimulation of pancreatic insulin secretion that leads to chronic hyperinsulinemia [2]. Hyperinsulinemia and insulin resistance may mediate cancer progression via the insulin/insulin-like growth factor axis [16]. Insulin and insulin-like growth factor 1 (IGF-1) are closely related hormones that control different aspects of growth and metabolism in many organisms [17,18]. Insulin receptor (IR) and IGF-1 receptor (IGF1R) transduce an intracellular signal primarily through the phosphatidylinositol 3-kinase (PI3K) pathway and the mitogen-activated protein kinase (MAPK) pathway [19]. It is known that IGF1R is involved in mitogenesis, transformation, and apoptosis protection [20,21]. In our previous work, the IGF-1 signal’s pivotal role in the onset of esophageal cancer was suggested in a duodenal-reflux-dependent esophageal carcinogenesis mouse model, even in the absence of hyperinsulinemic conditions [22].

Several studies have shown that a moderate calorie and/or protein restriction could reduce the risk of neoplastic disease development by bringing back the altered metabolic-hormonal status to a physiological condition and stimulating cell autophagy [23,24]. We performed a two-arm randomized clinical trial to assess the effect of a moderate calorie–protein restriction program (CARE-PRO TRIAL) on overweight or obese patients with Barrett’s esophagus. A similar approach has not been attempted in this disease context until now. Patients were divided randomly into two arms: a control arm (CA) and an intervention arm (IA). The control arm received information regarding the importance of a healthy lifestyle in reducing the risk of cancer, in accordance with the cancer prevention recommendations provided by the WCRF/AICR [14]. The intervention arm was involved in a two-year program of moderate caloric and protein restriction associated with moderate physical activity based on individual possibilities.

## 2. Materials and Methods

### 2.1. Trial Design

This was a single-center two-arm randomized controlled trial comparing overweight or obese BE patients undergoing CARE-PRO intervention with overweight or obese BE patients who only received general information about correct dietary habits and lifestyle, in accordance with the cancer prevention recommendations provided by the WCRF/AICR [14]. The intervention lasted 24 months and was accompanied by normal follow-up procedures [25] for both intervention and control patients. Briefly, participants in IA were provided with dietary advice based on the WCRF/AICR recommendations. Patients’ intake of total daily calories was reduced up to 600 kcal below their energy requirements, estimated using the revised Harris–Benedict formula [26]. Each patient was involved in sessions of culinary practice. Every session and every recipe were developed based on the Mediterranean Diet, in accordance with the WCRF/AICR recommendations and the Healthy Eating Plate proposed by the Harvard School of Public Health [27]. Participants in the CA received a leaflet based on the same recommendations [14] including suggestions to increase the consumption of fiber and vegetable proteins. The detailed intervention program is reported in Supplementary File S1. The research team, including research nurses, was blinded to the patient group allocation until the collection of laboratory data was finalized, except for the principal investigator, the dietitian, and participants, given the nature of the intervention. The latter three members of the research team were not involved in data analysis.

### 2.2. Study Population: Inclusion and Exclusion Criteria

BE patients considered for inclusion were those undergoing esophageal endoscopy surveillance at the Digestive Endoscopy Unit of the IOV-IRCCS. Participants’ confidential data were collected and held by the Digestive Endoscopy Unit. A detailed description of the intervention program is available at ClinicalTrials.gov (Registration Number NCT03813381).

The primary outcome of the study was to evaluate the impact of the lifestyle-oriented (diet, physical activity, and behavior change) intervention on (i) body mass index and waist circumference of overweight or obese BE patients. A 7% weight loss has been shown to be clinically effective in reducing the risk of diabetes [28]. Secondary outcomes were as follows: (ii) to determine the variation in the levels of serum analytes related to metabolic conditions associated with obesity (leptin and adiponectin), insulin resistance, and cancer risk (glycaemia, insulin, C-peptide, IGF-1, and IGF binding protein 3); (iii) to quantify the change of the esophageal expression level of the main proteins involved in insulin/IGF-1 signal transduction; (iv) to assess the modification of esophageal microbiota composition; (v) to evaluate adherence to the cancer prevention recommendations (WCRF/AICR 2007) [14] using a validated score [15]; and (vi) to evaluate possible differences in CARE-PRO outcomes between elderly (≥65 years) and younger BE patients (<65 years).

Inclusion criteria:

- Histological confirmation of Barrett’s esophagus without dysplasia or cancer;
- Aged ≥ 18 years;
- BMI ≥ 25.0 kg/m^2^;
- Willingness and ability to perform supervised Nordic walking sessions twice a month and self-planned physical activity at least three times a week;
- Signed informed consent.

Exclusion criteria:

- No histological confirmation of Barrett’s esophagus;
- Cancer diagnosis within one year prior to the trial’s commencement;
- Presence of insulin-dependent diabetes;
- Refusal to sign the informed consent.

Essential clinical data pertaining to analyzed patients from the control arm and intervention arm are shown in Table 1.

### 2.3. Sample Size

**Figure 1:**
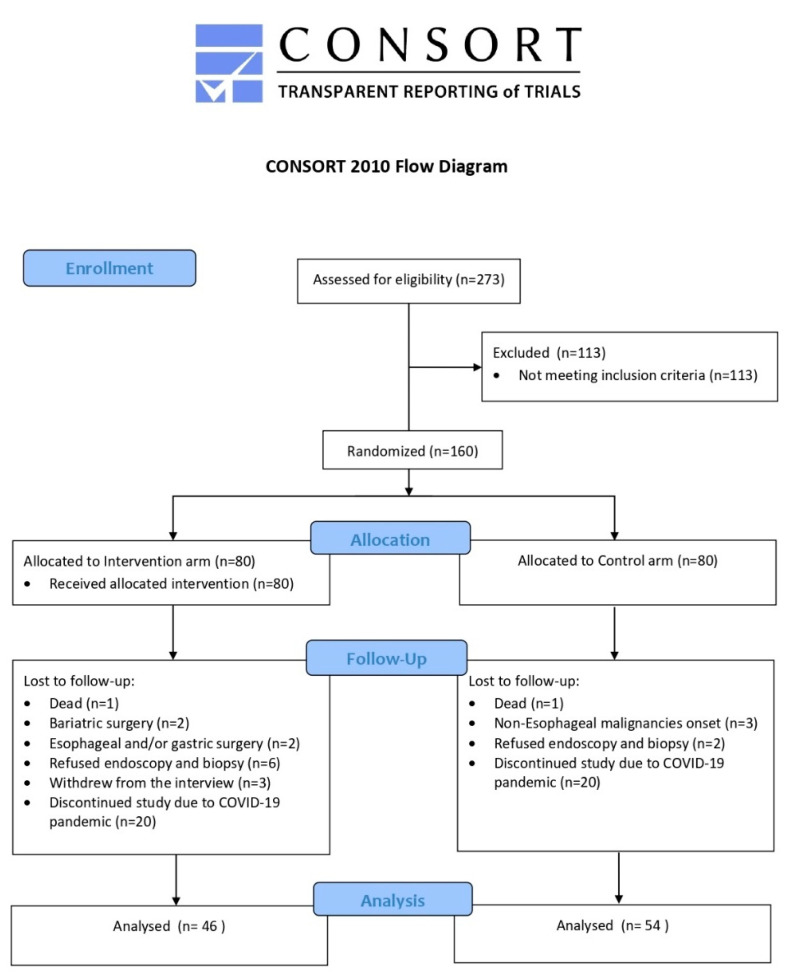
The trial’s CONSORT flow diagram.

The sample size was based on the primary outcome of a clinically significant change in body weight at the end of the study. A 7% weight loss has been shown to be clinically effective in reducing the risk of diabetes [28]. According to previous data [29], it is expected that around 30% of patients enrolled in the intervention arm and 15% of those enrolled in the control arm will achieve the weight loss within the time allotted for the trial. At least 95 patients were required for each group (power: 80%; type-1 error: 5%) in order to reveal a statistically significant difference in the rates of achievement of a 7% weight loss between IA and CA. It was estimated that a pool of 340 patients needed to be recruited to achieve this sample size. This would allow an enrollment rate of 70% (*n* = 238), and a subsequent dropout rate of 20% (*n* = 190). Due to the COVID-19 pandemic situation, the period of enrollment, which began in November 2015, was forced to close in March 2020. Until that date, a total of 273 patients with previously diagnosed BE underwent upper endoscopy in our unit at the Istituto Oncologico Veneto for endoscopic surveillance: 113 of them did not meet the inclusion criteria; 160 patients were randomized and allocated to the two arms as scheduled by the CARE-PRO study (IA *n* = 80; CA *n* = 80). Only patients enrolled up to February 2018 (*n* = 120, IA *n* = 60; CA *n* = 60) (able to complete the trial by February 2020) were included in this analysis. Excluding dropouts, the number of subjects included in the present analysis was n = 100 (IA *n* = 46; CA *n* = 54). The CONSORT flow diagram is shown in Figure 1.

### 2.4. Randomization

At the time of enrollment, prior to group allocation, anthropometric measurements (such as height, body weight, and waist circumference at the umbilical level) were recorded for each patient immediately before the endoscopic procedure. Patients were randomized to one of the two arms with a 1:1 allocation rate by the data manager, using a permuted block balanced procedure. The data manager was not involved in the recruitment or intervention process.

### 2.5. Blood and Biopsy Sample Collection

Immediately before endoscopy at both T0 and T24, blood samples were obtained from each patient (after 10–12 h of fasting) and blood glucose was measured with a glucometer (Abbott Laboratories^®^, Dallas, TX, USA). Serum was extracted, aliquoted, and frozen in liquid nitrogen. During endoscopy, 4 quadratic esophageal biopsies every 2 cm were obtained in line with the Seattle protocol, fixed in formalin, and sent to the Surgical Pathology Unit for histological examination to confirm the presence of Barrett’s esophagus and exclude the presence of dysplasia or cancer. Additional target biopsies were obtained from Barrett’s esophagus at 2 cm above the esophagogastric junction, and frozen in liquid nitrogen. Biopsies and serum samples were stored at −80 °C and managed by the Biological Bank staff who provided for their preservation in total anonymity (as prescribed by law and already authorized by the Ethics Committee with Prot. No. P 480 2002).

### 2.6. Serum Analysis

Serum metabolic biomarkers such as insulin, C-peptide, total IGF-1 and its binding protein 3, leptin, and adiponectin levels were simultaneously measured in each serum sample with Luminex xMAP^®^ technology (multiplexed fluorescent bead-based immunoassay) (Luminex Corporation, Austin, TX, USA). Each measure was performed in duplicate. Serum analyte quantitative analyses were performed with Luminex xPONENT 3.1 Software (Luminex Corporation, Austin, TX, USA) using a five parameter logistic curve fitting. The insulin resistance index (HOMA-IR index) was calculated using the formula: (fasting glucose (mg/dL) × fasting insulin (μU/mL)/405), according to the method developed by Matthews et al. [30]. The HOMA-IR index ≥2.5 was selected as the cutoff for insulin resistance according to Capasso et al. [31].

### 2.7. Esophageal Biopsy Analysis: Insulin/IGF-1 Signal Transduction

Total proteins were extracted from fresh frozen biopsies collected on distal esophageal segments. After mechanical lysis, tissues were treated with NP40 lysis buffer with added protease and phosphatase inhibitors (Thermo Scientific Pierce, Rockford, IL, USA). Bicinchoninic acid (BCA) assay (Thermo Scientific Pierce, Rockford, IL, USA) was performed to quantify extracted total proteins; 25 μg of total protein were analyzed for each sample and each analysis. The beta-tubulin amount was used as an internal loading control. Signaling pathways for total and phosphorylated IR/IGF1R proteins were determined using Luminex xMAP^®^ technology. Each measure was performed in duplicate. Both the metabolic PI3K/Akt pathway and the mitogenic (ERK1/2) pathway were analyzed. The quantification of a specific total protein was calculated as the ratio between each protein’s mean fluorescence intensity (MFI) and beta-tubulin MFI. Specific phosphorylated proteins were expressed as the ratio between each phosphorylated protein MFI and the total corresponding protein MFI. In the figures, data were expressed as median (Q1; Q3) and the relative protein expression was indicated as n-fold of the protein amount detected on the control arm measured at baseline (CA T0), which was set equal to 1 arbitrary unit (A.U.).

### 2.8. Statistical Analysis

The Mann–Whitney U test was used to assess differences between the two groups of patients at baseline. Fisher’s exact test was used for the comparison of categorical variables. The Wilcoxon matched-pairs signed-rank test was used to evaluate differences between T0 and T24 measurements within each group. Data were expressed as median and interquartile range (Q1; Q3). A *p*-value lower than 0.05 was assumed to indicate a significant difference. Data analyses were performed with STATA 12.0 (StataCorp. 2011. Stata Statistical Software: Release 12. College Station, TX: StataCorp LP, USA) and R software (version 3.5.1).

## 3. Results

### 3.1. Effect of the Intervention Program on Anthropometric Measurements

Anthropometric parameters (height (m), weight (kg), and abdominal circumference (cm)) were recorded at T0 and T24 for both IA and CA patients. Data on height and weight were used to calculate the body mass index (BMI). The Mann–Whitney U test results showed no differences between the two groups of patients at baseline (Table 2). The paired test showed no differences in anthropometric measurements from the time of enrollment to 24 months later in CA patients. To the contrary, patients in the IA group showed a significant decrease in body weight (*p* = 0.001) and, as a consequence, in BMI measurement, with a median reduction of Δ = −0.84 kg/m^2^ (*p* = 0.001). Similarly, in the same group of patients, waist circumference (WC) values decreased after 24 months (*p* = 0.002) (Table 2).

The overall median percentage of the weight loss in the intervention arm was 3.0%. Weight loss and BMI reduction were found in 35 IA patients out of 46 (76.1%) and 19 CA patients out of 54 (35.2%). The Fisher’s exact test showed a significant difference between the groups (*p* < 1 × 10^−4^). Seven out of 46 IA patients achieved a reduction in body weight at least equal to 7%. Conversely, no patient included in the control arm achieved this goal (Fisher’s exact test, *p* = 0.003). Regardless of the extent of WC reduction, values decreased in 69.6% and 48.1% of IA and CA patients, respectively (Fisher’s exact test, *p* = 0.042). At the time of enrollment, 94.4% of CA patients (*n* = 51) and 89.1% of IA patients (*n* = 41) (*p* = 0.465) were affected by central obesity (i.e., waist circumference values ≥94 cm and ≥80 cm, for European males and females, respectively, according to the International Diabetes Federation definition [32]). At the end of the program, 2.0% of CA patients (1 out of 51) and 21.9% of IA patients (9 out of 41) affected by central obesity achieved a decrease in waist circumference up to the normal value (Fisher’s exact test, *p* = 0.004). A reduction of both WC and BMI was observed in 65.2% of IA patients and 22.2% of CA patients (*p* < 1 × 10^−4^).

These data suggest that providing information about the correct lifestyle and dietary habits to be adopted to prevent the evolution of disease can produce positive effects on about 20% of informed people. On the other hand, the substantial changes observed in 65% of patients actively involved in the intervention program suggest the effectiveness of the proposed multimodal nutritional intervention (intervention program details are summarized in Supplementary File S1: Intervention Program, or fully reported at ClinicalTrials.gov (accessed 2021 -10 -17).

### 3.2. Effect of the Intervention Program on Serum Parameters

In order to evaluate each patient’s metabolic status at enrollment, fasting blood glucose, fasting serum insulin, C-peptide, IGF-1, its binding protein 3, leptin, and adiponectin levels were measured. The HOMA-IR index was calculated to estimate insulin resistance conditions. No differences were observed at baseline between CA and IA for each parameter considered (as indicated by Mann–Whitney U test results reported in Table 3). The same parameters were measured after 24 months.

A significant reduction in IGF-1 levels was observed in IA patients at the end of the intervention program, with a median value of variation (Δ) = −0.91 nmol/L (11.2% lower than basal values measured at the time of enrollment). Analysis showed no significant differences in the other serum parameters considered.

Although mild in magnitude, significant increases in fasting glucose (Δ= +2.0 mg/dL, 2.6% higher than basal level) and serum leptin levels (Δ= +0.98 ng/mL, 15.4% higher than basal level) were observed in CA patients. The adipokine ratio (leptin/adiponectin) was also significantly increased in the CA group (Δ= +0.10 ng/μg, 50.0% higher than baseline), while IGFBP3 serum levels were lower (–6.3%) compared to basal levels (Δ= −1.0 nmol/L).

At the time of enrollment, 37.0% of CA patients and 36.9% of IA patients were insulin resistant (HOMA-IR index ≥ 2.5). At the end of the intervention, 20.0% of CA and 17.6% of IA insulin-resistant patients returned to normal values (*p* = 0.701) (HOMA-IR index < 2.5). Conversely, after 24 months from enrollment, 14 CA patients and 1 IA patient with a normal HOMA-IR index at T0 increased their HOMA-IR index to reach values attributable to an insulin resistance condition. The Fisher’s exact test result indicated significant differences between the groups (*p* = 7 × 10^−4^).

**Figure 2:**
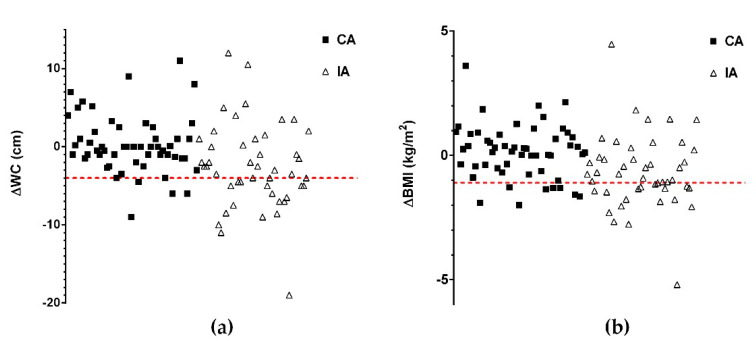
Waist circumference variation (ΔWC) and BMI (ΔBMI) in the control arm (CA) and intervention arm (IA) at the end of the 24 months of the intervention program. (**a**) ΔWC and (**b**) ΔBMI were calculated as the difference between the value measured at T24 and the baseline value. The dashed red lines (ΔWC = −4 cm and ΔBMI = −1.11 kg/m^2^) show the cutoffs between the two arms, which were mathematically corresponding to the 25th percentile of the whole data distribution. Each square represents a single patient from the CA and each triangle represents a single patient from the IA.

Most of the patients with a higher reduction in waist circumference (WC reduction ≥ 4.0 cm) (Figure 2a) were in the IA group (20 out of 26 patients), while just 6 patients were part of the CA (*p* = 5 × 10^−4^). Likewise, most of the patients with a higher reduction in BMI values (BMI reduction ≥ 1.11 kg/m^2^) belonged to the IA (17 out of 25 patients), while only 8 patients were from the CA group (*p* = 0.019) (Figure 2b). Patients who satisfied the identified cutoffs had also improved in a higher number of serum parameters (an average of about 6 out of 10 parameters reported in Table 3) compared with the rest of the patients (an average of about 4 parameters) The Mann–Whitney U test showed significant differences in the number of improved serum parameters (*p* = 0.008 and *p* = 0.004 for WC and BMI variations, respectively). The relationship between anthropometric and serum metabolic parameters suggests that the improvements were associated with significant weight loss and significant central adiposity reduction. In accordance with the improvement of anthropometric parameters, more frequently observed in the IA, the most positive changes in serum metabolic factors were found among patients actively involved in the supervised intervention program.

### 3.3. Effect of the Intervention Program on the Insulin/IGF-1 Signal in Esophageal Biopsy Specimens

The expression and activation state of the main proteins involved in the insulin/IGF-1 signaling pathway (listed in Table 4) were measured in bioptic samples obtained from the distal esophagus of BE patients during the upper endoscopies required by the protocol.

**Figure 3:**
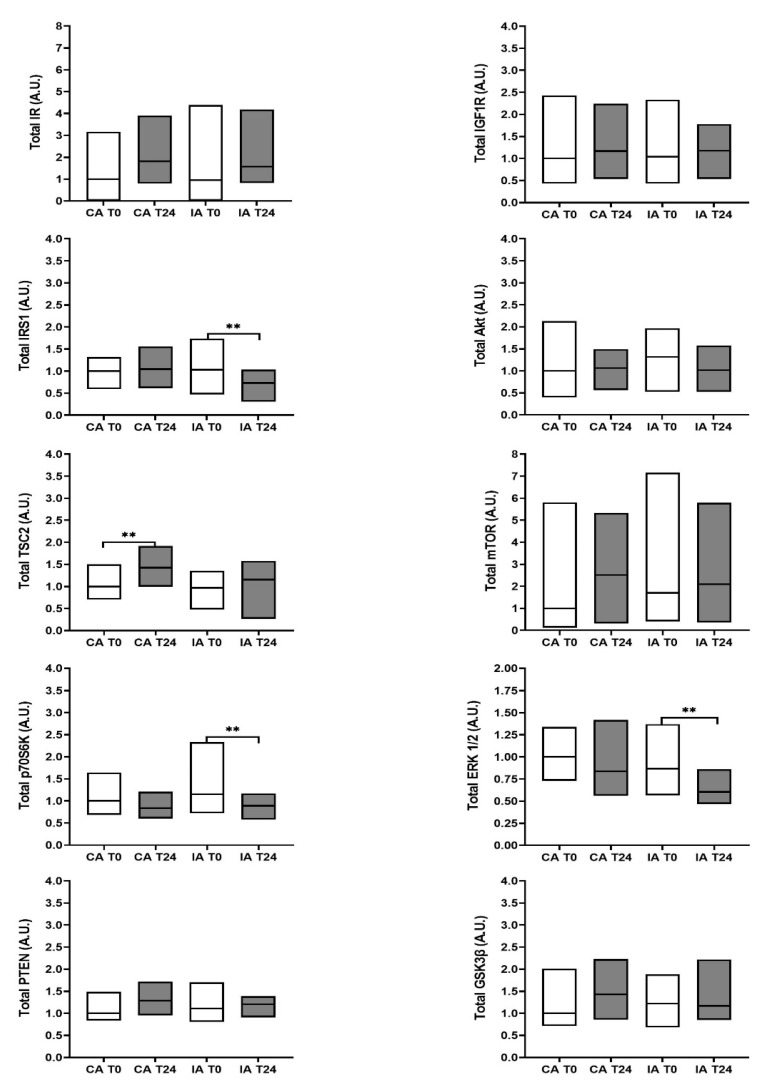
Total protein expression involved in insulin/IGF-1 signal transduction in esophageal tissue affected by Barrett’s esophagus. Protein expression was analyzed at baseline (T0, white boxes) and 24 months later (T24, black boxes) in both the control arm (CA) and intervention arm (IA). Data are expressed as the median (Q1; Q3) related to an arbitrary unit (A.U.), defined as described in the Materials and Methods Section. The Wilcoxon matched-pairs signed-rank test was used to compare measurements at T0 and T24 within the same group. The Mann–Whitney U test was performed to evaluate differences between the CA and IA at baseline. ** *p* < 0.01.

None of the evaluated protein expressions showed significant differences between the two groups at the time of enrollment (Figure 3). Total protein expression in the CA esophageal tissue was tested and showed no statistically significant differences between the two time-points considered, except for TSC2 protein levels. At time T24, TSC2 was overexpressed 1.42-fold higher than baseline values (*p* = 0.005) (Figure 3).

**Figure 4:**
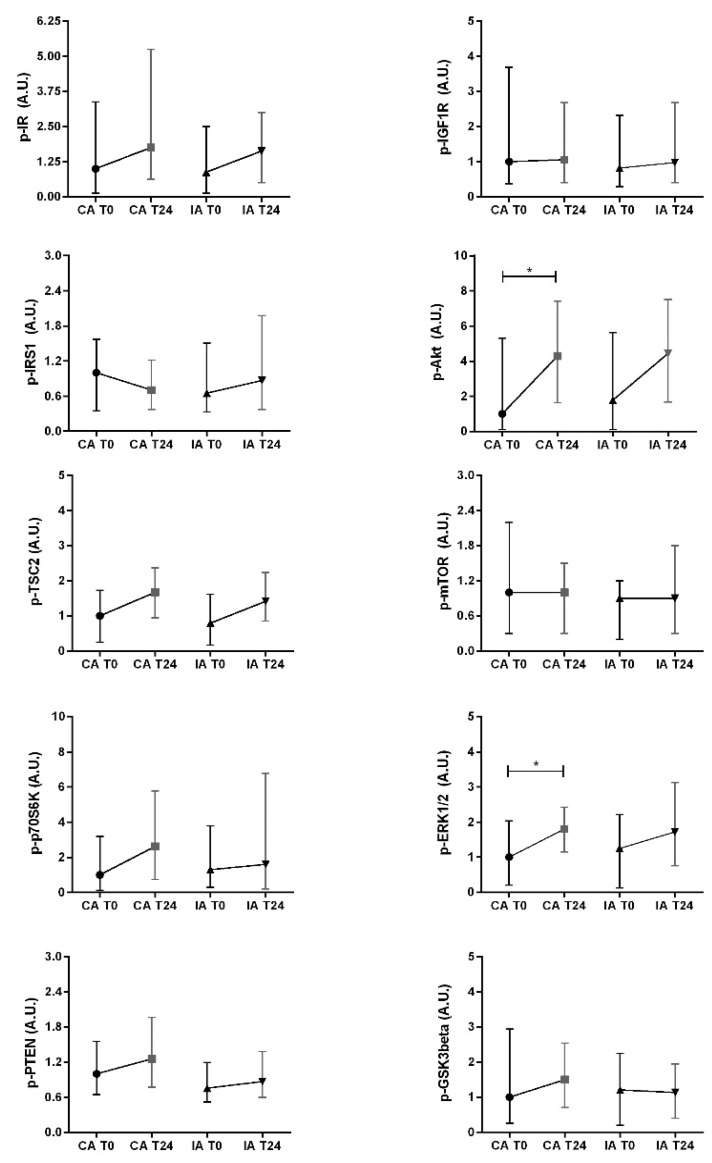
Main protein phosphorylation levels in insulin/IGF-1 signal transduction in esophageal tissue affected by Barrett’s esophagus. Protein expression was analyzed at baseline (T0) and 24 months later (T24) in both the control arm (CA) and the intervention arm (IA). Data are expressed as the median (Q1; Q3) of relative phosphorylation levels related to an arbitrary unit (A.U.) defined as described in the Materials and Methods Section. Each symbol represents a different data set: ● CA at T0, ∎ CA at T24, ▲ IA at T0, ▼IA at T24. The Wilcoxon matched-pairs signed-rank test was used to compare measurements at T0 and T24 within the same group. The Mann–Whitney U test was performed to evaluate differences in baseline measurements between the CA and IA. * *p* < 0.05.

In terms of the phosphorylation state of the proteins in the CA group, patients showed a significant increase in the relative phosphorylation of both the Akt and ERK1/2 (paired test, *p* = 0.031 and *p* = 0.018, respectively) (Figure 4).

In contrast, several differences were observed in the total protein expression levels of the IA group between T0 and T24 (Figure 3). The total IRS1 expression level at T24 was reduced by 23.20% (*p* = 0.006). Furthermore, the total p70S6K and total ERK1/2 expression decreased significantly in IA precancerous tissue after 24 months (paired test, *p* = 0.003 and *p* = 0.003, respectively). The median downregulated expression was 27.86% for p70S6K and 22.87% for ERK1/2. No differences were observed in the other total proteins analyzed. The analysis of the relative protein phosphorylation level in the IA group showed no significant variation (Figure 4).

### 3.4. Overall Effect of the Intervention Program: Identification of Different Patient Subpopulations

The unexpected downregulation of the IRS1 protein in patients with significant weight loss and reduced abdominal fat prompted us to perform further analyses. IRS1 downregulation was observed in 31 out of 46 patients in the IA (67.4%) and 22 out of 54 in the CA (40.7%) (53 patients were identified as the lower-IRS1 subpopulation).

**Figure 5:**
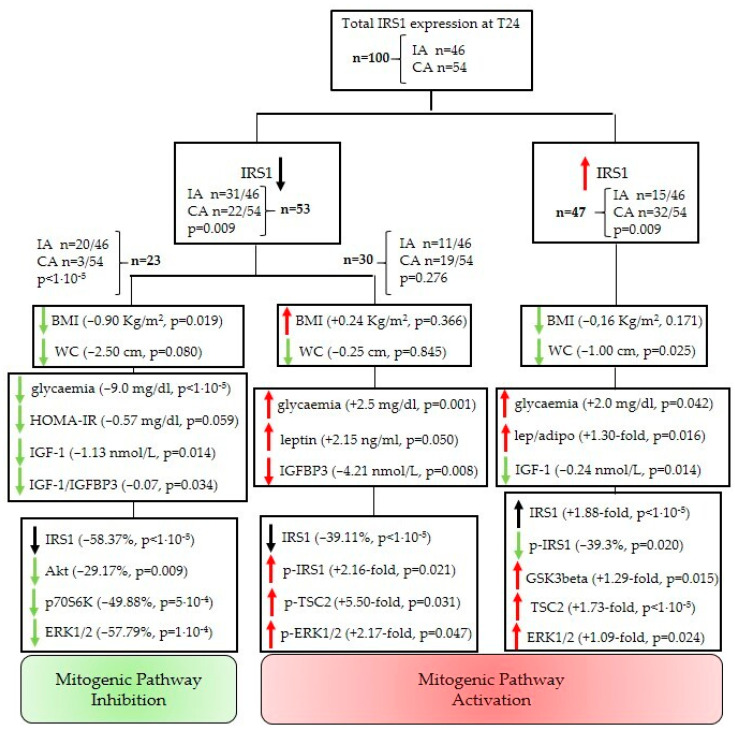
Identification of different patient subpopulations based on the modulation of IRS1 expression after 24 months from the start of the intervention program. Patients were divided on the basis of the downregulation (lower) or upregulation (higher) of IRS1 expression at the end of the intervention program compared to the basal expression at the time of enrollment. Within the lower IRS1 subpopulation, patients who had improved glycaemic control and patients who had unchanged or worsened glycaemic control were analyzed separately. For each group of patients, data regarding anthropometric parameters, serum analytes, total expression, and the relative phosphorylation level of proteins extracted from Barrett’s lesion were reported. Data indicate the median variation (T24−T0) of the parameter considered, followed by a *p*-value (the Wilcoxon matched-pairs signed-rank test was used to compare measurements at T0 and T24 within the same group). A reduction in protein levels was indicated as the median of the variation expressed as a percentage (%). An increase in protein level was indicated as n-fold of the amount detected at baseline. Parameters not included in this diagram were not significantly modified. ^1^ The Fisher’s exact test was performed to assess differences between the proportions of patients belonging to the two arms within each subpopulation identified. The up/down arrow indicates an increase/decrease in the parameter’s measure at 24 months compared to baseline. The green arrow indicates a positive change (protective against cancer evolution) in the parameter considered. The red arrow indicates a negative change (procancer) in the parameter considered.

The analysis of the parameters in the lower-IRS1 subpopulation allowed us to identify two different subpopulations: the first subpopulation showed a significant BMI reduction (median reduction = −0.90 kg/m^2^, z = −2.34, *p* = 0.019), and lower levels of several serum parameters. Indeed, blood glucose levels were reduced (z = −4.20, *p* < 1 × 10^−5^), as well as IGF-1 levels (z = −2.46, *p* = 0.014) and the IGF-1/IGFBP3 ratio (z = −2.11, *p* = 0.034). The HOMA-IR index variation was close to significance (z = −1.89, *p* = 0.059). Figure 5 shows the median variation of each analyte considered. The same subpopulation, together with IRS1 downregulation (z = −4.20, *p* < 1 × 10^−5^) in esophageal tissue affected by Barrett’s lesion, importantly showed a decreased amount of total Akt (z = −2.62, *p* = 0.009), total p70S6K (z = −3.47, *p* = 5 × 10^−4^), and total ERK1/2 protein (z = −4.04, *p* = 1 × 10^−4^). Twenty-three patients in total (*n* = 20 IA and *n* = 3 CA patients) belonged to this lower-IRS1 and improved metabolic parameters group (Figure 5).

To the contrary, the second subpopulation showed no weight loss (z = +0.90, *p* = 0.366), an increase in blood glucose level (z = +3.30, *p* = 0.001), a significant increase in leptin secretion (z = +1.95, *p* = 0.050), and an important reduction in IGFBP3 (z = −2.64, *p* = 0.008). This population, together with esophageal IRS1 downregulation, showed a significant increase in the phosphorylation of proteins analyzed: IRS1 relative phosphorylation (z = +2.31, *p* = 0.021), TSC2 relative phosphorylation (z = +2.15, *p* = 0.031), and ERK relative phosphorylation (z = +1.98, *p* = 0.047). Thirty patients in total (*n* = 11 IA and *n* = 19 CA patients) belonged to this lower-IRS1 and worsened metabolic parameters group (Figure 5).

Taken together, the results suggest that in patients who actively followed the program, the reduction in BMI and WC induced recovery in insulin sensitivity, with a lower IGF-1 serum level. A significant downregulation of most proteins involved in insulin-induced molecular signal transduction occurred as a result of the new metabolic state. The downregulation of the signal seems to involve intracellular cascade proteins such as IRS1, p70S6K, and ERK total expression. The simultaneous downregulation of the main proteins involved in insulin/IGF-1 molecular signal transduction occurred mainly in IA patients and it was related to weight loss and an improvement in glucose homeostasis. Notably, only 3 out of 54 of the CA patients achieved this condition at the end of the trial.

The downregulation of total IRS1 was not exclusively associated with an improvement of the metabolic parameters. In fact, in a different lower-IRS1 subpopulation, 63.3% of whom belonged to the CA, this alteration was found and occurred simultaneously with the increase in IRS1 phosphorylation in serine residues, with reduced activity of the tumor suppressor TSC2 and increased activity of ERK proteins. These molecular adjustments suggest a reduction in insulin sensitivity, which was confirmed by a worsening of glycaemic control associated with an increase in leptin secretion.

The remaining patients, who showed IRS1 upregulation in their esophageal tissue at the end of the 24-month intervention program (the higher-IRS1 subpopulation), shared some characteristics with both the populations described above, in particular, reduced IGF-1 secretion (z = −2.46, *p* = 0.014) similar to the first population and a significant increase in blood glucose levels (z = +2.03, *p* = 0.042) similar to the second. A negligible WC reduction was observed (z = −2.11, *p* = 0.035), but it was insufficient to make improvements in the regulation of adipokine release (the leptin/adiponectin ratio was 1.30-fold higher than baseline, z = +2.40, *p* = 0.016). In addition to an increase in IRS1 total expression (z = +5.97, *p* < 1 × 10^−5^), a significant increase in ERK1/2 expression (z = +2.25, *p* = 0.024) was observed. Analogous with data observed in the whole group of CA patients (Figure 3), this population showed a higher expression of TSC2 in esophageal tissue (z = +4.57, *p* < 1 × 10^−5^). Unlike the other populations, here we observed a significant reduction in the relative inhibitory phosphorylation of IRS1 (z = −2.33, *p* = 0.020) and an increase in GSK3-beta total expression (z = +2.42, *p* = 0.015). A total of 47 patients (*n* = 15 IA and *n* = 32 CA patients) were in this higher-IRS1 group (Figure 5).

These data suggest that a small reduction in abdominal fat accumulation was insufficient to reverse the deficiency in the glucose homeostasis mechanism. Furthermore, a small decrease in IGF-1 secretion could not be sufficient to inhibit the mitogenic mechanism, as was suggested by the increase in ERK1/2 expression in esophageal tissue.

## 4. Discussion

Central obesity, intrinsically associated with insulin resistance and hyperinsulinemia, has been previously identified as an important risk factor for the development of esophageal adenocarcinoma [5,6,7]. The main mechanisms involved include anatomic factors such as increased abdominal and intraperitoneal fat accumulation, as well as diseases associated with predisposing patients to increased gastroesophageal reflux, such as hiatal hernia formation. Moreover, obesity may mediate cancer progression through insulin resistance via the insulin/insulin-like growth factor axis, oxidative stress, chronic systemic inflammation, and dysregulated adipokines secretion [2,33,34]. Our group previously reported how hyperinsulinemia can induce hyperactivation of esophageal receptors for insulin and IGF-1, and how their impairment and the commitment of other growth factor receptors, such as HER2, can mediate insulin’s pro-proliferative effect on precancerous and cancerous esophageal cells [5]. The pivotal role of IGF1R signaling in esophageal cancer onset has been exhaustively studied in our surgically induced esophageal adenocarcinoma mouse model. Our data suggest the involvement of IGF1R in esophageal carcinogenesis, even in the absence of hyperinsulinemia or obesity conditions [22]. Nutrient excess, together with genetic predisposition, may play a crucial role in the development of insulin resistance. Increased fat intake is considered to be the main cause of metabolic abnormalities related to overnutrition. Although short-term trials showed that high-protein diets have some beneficial effects on glucose homeostasis in obese and diabetic patients [35,36], there is rising scientific evidence suggesting that elevated dietary protein consumption (particularly of animal origin) is associated with increased insulin resistance, glucose intolerance, and the risk of type 2 diabetes [37]. Long-term nutritional approaches, based only on calorie restriction or protein restriction, showed beneficial effects on insulin and inflammatory mediators in humans. Exclusive protein restriction was able to induce a reduction in total and free IGF-1 levels [38], suggesting that reduced protein intake could represent an important component of anti-cancer dietary interventions.

To our knowledge, the present study is the first to investigate the effect of a moderate calorie and protein restriction program on overweight or obese patients affected by the esophageal precancerous lesion called Barrett’s esophagus. The effect was evaluated on the anthropometric parameters and levels of serum analytes related to insulin resistance. Moreover, the insulin/IGF-1 signaling pathway was analyzed in distal esophageal biopsy specimens obtained during an upper endoscopy. All the parameters were analyzed at the time of enrollment and 24 months later in patients who underwent the active multimodal restriction program, and in the control group who only received a leaflet based on the WCRF/AICR recommendations [14]. Data on anthropometric parameters suggest that just providing written information about the correct lifestyle and dietary habits to be followed can produce positive effects on around 20% of informed people. Instead, the meaningful changes observed in approximately 65% of patients from the IA group suggest that the effectiveness of the nutritional intervention is significantly increased when specialized personnel, besides providing information, use health coaching techniques, give patients the opportunity to practice acquired knowledge, and plan group activities, such as cooking classes, sharing meals, and collective involvement in physical activity.

The new lifestyle and dietary habits seem to be appropriate for reducing IGF-1 secretion. The relation between anthropometric and serum metabolic parameters suggests that most of the improvements were related to significant weight loss associated with a significant decrease in central adiposity. In particular, the cutoff assigned to a reduction in waist circumference (4 cm) and BMI (1.11 kg/m^2^) was indicative of the endpoint for obtaining beneficial metabolic changes. According to the improvements observed in BMI and WC, the most positive changes in serum metabolic factors were found among patients actively involved in the supervised intervention program. In particular, adherence to the program revealed a double positive effect: on the one hand, it improved insulin resistance conditions and, on the other, could prevent possible deterioration, as was suggested by the control arm trend, particularly for glycaemia, and HOMA-IR index changes, and the reduction in the IGF-binding protein 3 in these unsupervised patients.

While lifestyle intervention was able to produce enough weight loss to improve serum metabolic parameters, it is important to consider which parameters to alter and the range to which they must be changed, in order to induce an appreciable improvement in insulin/IGF-1 signaling directly on esophageal tissue affected by Barrett’s lesion. Data obtained on esophageal protein expression show evidence of a new metabolic state. This state is associated with a significant downregulation of most proteins involved in insulin/IGF-1-induced molecular signal transduction in the BE patients participating in the intervention program. The analysis of the whole IA group showed a reduced amount of IRS1, p70S6K, and the extracellular signal-regulated kinase (ERK1/2) total protein expression in esophageal BE tissue, accompanied by a significant reduction in IGF-1 serum level.

The unexpected downregulation of expression of the first substrate recruited by insulin and IGF-1 activated receptors prompted us to investigate further. We observed that patients showing reduced expression of IRS1 belonged to two distinct populations: the first with IRS1 downregulation exhibited a significant decrease in expression of the other main proteins involved in insulin/IGF-1 signal transduction, such as Akt, p70S6K, and ERK1/2. These patients lost body weight, showed reduced glycaemia, an improved HOMA-IR index, decreased IGF-1 serum levels, and a lower IGF-1/Binding protein 3 molar ratio. This subpopulation was composed of 23 patients, 87.0% of whom were from the intervention arm. Data suggest that glucose homeostasis and insulin sensitivity improved in these patients. Their metabolic response to the change in dietary habits, coupled with moderate physical activity, was able to induce molecular modifications of the insulin/IGF-1 pathway in the esophageal tissue affected by precancerous lesions. This group fully benefited from the interventional approach that finally resulted in the inhibition of the mitogenic pathway in precancerous lesions.

The second subpopulation with IRS1 downregulation showed a significant increase in relative inhibitory phosphorylation of IRS1 and TSC2 anti-tumor protein and an increased activation of the mitogenic pathway associated with ERK1/2. These patients were characterized by no weight loss, increased levels of blood glucose and serum leptin, and a decreased level of serum IGF-binding protein 3. This subpopulation was composed of 30 patients, 63.3% of whom were from the control arm. The worsening of glucose homeostasis and the increase in free IGF-1 were the result of the lowest insulin sensitivity mediated by the IRS1 downregulation. As is known, IRS1 phosphorylation on serine residues precedes its degradation. The phosphorylation is mediated by S6K1, an insulin-induced serine/threonine kinase that negatively modulates the effect of insulin [39]. These data highlight that this group did not reap any benefits from the program due to a worsening of their metabolic condition that continued to induce the activation of the mitogenic pathway.

Additionally, we identified a third group with a high IRS1 expression composed of 32 patients (59.2%) enrolled in the control arm and 15 IA patients (32.6%) (higher-IRS1 subpopulation). This group had a marginal metabolic change that could be the consequence of a negligible reduction in abdominal fat; it was manifested by a modest reduction in IGF-1 secretion, curiously accompanied by a more marked alteration in adipokines secretion. Furthermore, increased GSK3-beta levels were detected in these patients. This could be responsible for the inhibition of glycogen synthesis [40], accompanied by a small increase in glycaemia. The modification of metabolic conditions could be involved in the activation of mitogenic pathways in these patients. The upregulation observed for the TSC2 protein could be explained by the necessity to limit and control the events responsible for cell transformation [41]. As previously reported by Ma et al. [42], the main cellular function of TSC2 is to inhibit the S6K1 protein. This fact could explain the reduced relative phosphorylation of IRS1, a specific target of S6K [43]. This population involved patients who achieved few benefits and, therefore, the outcomes do not reflect a substantial lifestyle change that can mitigate the activation of the mitogenic pathway.

IGF-1 is a potent mitogenic growth factor. It promotes cell proliferation and inhibits apoptosis. We previously demonstrated the pivotal role played by IGF1R signaling in the onset of esophageal cancer in normo-insulinemic and hyperinsulinemic surgically-induced EAC mice [22]. Experimental and epidemiological studies suggest that IGF-1 and its binding protein serum levels are influenced by dietary intake, and play a role in the pathogenesis of several common cancers. Data from randomized clinical trials have shown that calorie restriction does not reduce IGF-1 and IGF-1/IGFBP3 ratio levels, unless protein consumption is also reduced [38]. Data from our 24-month randomized trial on patients affected by Barrett’s esophagus suggest that a calorie and protein restriction program together with a specialized supervised approach can affect IGF-1 serum levels, reducing its secretion and also the IGF-1/IGFBP3 ratio. This study also suggests that a significant reduction in IGFBP3 synthesis could occur independently of a significant alteration of total IGF-1 levels. A decrease in IGFBP3 is a remarkably important index that underlines the worsening of the metabolic condition and tissue procancer molecular changes. An interesting amount of evidence points to the IGF-independent effects of IGFBP3 on cell growth. This protein acts as a growth inhibitor and tumor suppressor [44]. Data from our previous retrospective study suggested a role for reduced IGFBP3 secretion in both Barrett’s onset and the evolution toward dysplasia/adenocarcinoma [5].

When IGF-1 secretion is lower and reflects a lower free IGF-1 concentration, confirmed by a decrease in the IGF-1/IGFBP3 ratio, combined with glycaemia reduction and weight loss, observed in the subpopulation mostly represented by IA patients, the metabolic condition improves enough to induce substantial positive molecular changes in the esophageal tissue affected by pretumor lesions. Adding total IGF-1 and the IGFBP3 to the routine blood sample control could represent an inexpensive and preventive approach to monitoring the metabolic condition trend of the patient.

Our study has some limitations. We excluded patients who were unable to complete the trial because of the COVID-19 pandemic. In fact, many of the programmed activities required for the intervention arm could not be carried out. For this reason, the sample size calculated and reported by the protocol has not been reached. This study includes the analysis of a large number of parameters. This manuscript’s focus is on the relationship between weight loss and the metabolic changes that can be measured in serum. The next step was to identify which parameters were significantly associated with the molecular changes that were observed directly in esophageal lesions. All the other aspects of this study, fully reported in the registered protocol, will be analyzed and commented on separately.

## 5. Conclusions

The fundamental objective to consider in a lifestyle change program should be the improvement of insulin sensitivity. To achieve this goal, acting only on lower calorie and protein assumptions is not sufficient: the decreasing intake of both calories and protein, the professional intervention of a dietician who attends each follow-up control, the cooking classes, and moderate physical activity based on the individual characteristics, are all important parts of the proposed intervention. Patients who responded optimally to the approach significantly improved glycaemic control, insulin resistance, and reduced IGF-1 availability, controlling and reducing the serum concentration of markers associated with increased risk of Barrett’s esophagus evolution towards adenocarcinoma. These important changes prompt a significant downregulation of the insulin/IGF-1 pathway, which results in a downregulation of the ERK-mediated mitogenic pathway directly at the esophageal tissue level in Barrett patients. At the end of 24 months, no patients involved in this study developed esophageal dysplasia or cancer. In order to evaluate the effectiveness of our preventive approach on esophageal adenocarcinoma, the long-term evolution of the disease will be monitored and associated with the parameters considered in this study.

## Figures and Tables

**Table 1:** Characteristics of the population.

	CA	IA
n (female)	54 (5)	46 (8)
Age, mean (±SD)	59.1 (±10.6)	58.7 (±10.1)
PPI/anti-acid n (%)	54 (100)	46 (100)
NSAID n (%)	24 (44.4)	18 (39.1)
Thyroid hormone replacement therapy n (%)	4 (7.4)	6 (13.0)
Anti-hypertensive therapy n (%)	25 (46.3)	16 (34.8)

Fisher’s exact test showed no differences between groups. All patients were Caucasians; n: number; SD: standard deviation; CA: control arm; IA: intervention arm.

**Table 2:** Anthropometric measurements at baseline (T0) and at the end of 24 months of intervention (T24).

	CA *n* = 54				IA *n* = 46		
	T0	Δ(T24−T0)	Paired	M–W	T0	Δ(T24−T0)	paired
Weight (kg)	84.30 (77.93; 94.80)	+0.35 (−1.58; +2.00)	z = +0.71 *p* = 0.477	0.767	84.95 (78.50; 92.75)	−2.55 (−4.08; −0.28)	z = −3.31 *p* = 0.001
BMI (kg/m^2^)	28.08 (26.55; 30.65)	+0.20 (−0.50; +0.81)	z = +1.01 *p* = 0.312	0.772	28.04 (26.25; 29.56)	−0.84 (−1.34; −0.09)	z = −3.21 *p* = 0.002
WC (cm)	103.75 (97.63; 109.75)	+0.00 (−1.50; +1.68)	z = −0.25 *p* = 0.799	0.070	101.00 (94.63; 106.95)	−2.75 (−5.00; +0.80)	z = −3.13 *p* = 0.001

Δ(T24−T0) indicates the difference between the value measured at T24 and the baseline value. Data are expressed as median (Q1; Q3). The Wilcoxon matched-pairs signed-rank test (paired) was used to compare measurements at T0 and T24 within the same group. The Mann–Whitney U test (M–W) was performed to evaluate differences in baseline measurements (T0) between the control arm (CA) and intervention arm (IA).

**Table 3:** Serum metabolic parameters at baseline (T0) and at the end of 24 months of intervention (T24).

	CA				IA		
	T0	Δ(T24−T0)	Paired	M–W	T0	Δ(T24−T0)	Paired
Glucose (mg/dL)	85.5 (77.0; 96.7)	+2.0 (−2.7; +4.7)	z = +2.16 *p* = 0.031	0.936	89.0 (77.0; 99.0)	−2.5 (−10.5; +3.5)	z = −1.01 *p* = 0.314
Insulin (pg/mL)	413.4 (282.6; 576.0)	+26.0 (−117.1; +131.2)	z = +0.60 *p* = 0.547	0.638	318.2 (207.2; 531.7)	−37.9 (−103.6; +66.8)	z = −1.16 *p* = 0.245
C-peptide (pg/mL)	1300.5 (840.2; 1650.8)	+4.1 (−215.4; +317.9)	z = +0.73 *p* = 0.464	0.254	1064.8 (772.1; 1448.5)	−55.7 (−205.9; +155.5)	z = −0.63 *p* = 0.530
HOMA-IR	2.20 (1.47; 3.37)	+0.23 (−0.45; +0.96)	z = +1.47 *p* = 0.142	0.110	1.75 (0.97; 3.26)	−0.29 (−0.74; +0.40)	z = −1.28 *p* = 0.199
IGF-1 (nmol/L)	8.33 (6.61; 10.85)	−0.11 (−1.89; +1.10)	z = −1.67 *p* = 0.095	0.219	9.65 (7.80; 11.56)	−0.91 (−3.15; +0.17)	z = −3.15 *p* = 0.002
IGFBP3 (nmol/L)	24.74 (19.57; 27.77)	−1.00 (−4.33; +1.10)	z = −2.17 *p* = 0.029	0.254	25.99 (22.60; 29.23)	+0.52 (−4.21; +2.21)	z = −0.55 *p* = 0.585
IGF-1/IGFBP3 molar ratio	0.37 (0.27; 0.44)	+0.01 (−0.61; +0.10)	z = +0.97 *p* = 0.333	0.834	0.35 (0.28; 0.44)	−0.03 (−0.13; +0.07)	z = −1.67 *p* = 0.096
Leptin (ng/mL)	6.08 (3.37; 10.75)	+0.97 (−0.89; +2.09)	z = +2.18 *p* = 0.029	0.153	5.17 (2.61; 8.15)	+0.02 (−1.72; +1.19)	z = +0.20 *p* = 0.844
Adiponectin (µg/mL)	14.50 (10.02; 25.56)	−1.08 (−7.81; +1.71)	z = −1.71 *p* = 0.087	0.529	18.92 (10.65; 25.45)	+0.41 (−3.02; +6.22)	z = +0.46 *p* = 0.642
Leptin/ Adiponectin	0.37 (0.23; 0.88)	+0.10 (−0.04; +0.39)	z = +3.10 *p* = 0.002	0.230	0.30 (0.12; 0.66)	−0.01 (−0.12; +0.12)	z = +0.02 *p* = 0.987

Δ(T24−T0) indicates the difference between the value measured at T24 and the baseline value. Data are expressed as median (Q1; Q3). The Wilcoxon matched-pairs signed-rank test (paired) was used to compare measurements at T0 and T24 within the same group. The Mann–Whitney (M–W) U test was performed to evaluate differences on baseline measurements (T0) between the control arm (CA) and intervention arm (IA).

**Table 4:** Proteins involved in the insulin/IGF-1 signaling pathway, whose expression and phosphorylation level were analyzed in esophageal biopsy specimens from Barrett’s lesion.

Total Protein	Phosphorylated Residue	Phosphorylation Function
IR	Tyr1162/Tyr1163	activating
IGF1R	Tyr1135/Tyr1136	activating
IRS1	Ser636	inhibitory
Akt	Ser473	activating
mTOR	Ser2448	activating
p70S6K	Thr412	activating
PTEN	Ser380	inhibitory
ERK1/2	Thr185/Tyr187	activating
GSK3-beta	Ser9	inhibitory
TSC2	Ser939	inhibitory

Total protein expression and phosphorylation levels were analyzed with Luminex xMAP^®^ technology. Phosphorylation on specific residues promotes an active protein conformation (activating). Conversely, some phosphorylation events negatively regulate protein activity (inhibitory).

## Supplementary Materials

The following are available online at https://www.mdpi.com/article/10.3390/nu13103638/s1, Supplementary File S1: Intervention Program.
