# Supplementary material for: Insulin/IGF-1 Signaling Is Downregulated in Barrett’s Esophagus Patients Undergoing a Moderate Calorie and Protein Restriction Program: A Randomized 2-Year Trial
Source: Nutrients. 2021 Oct 17;13(10):3638. doi: 10.3390/nu13103638 (PMC8537306; doi:10.3390/nu13103638)
Supplement: Supplementary file 1 [file nutrients-13-03638-s001.zip › nutrients-1408458-supplementary.pdf]

Supplementary File S1: Intervention Program

Control Arm (CA) Program

Participants in the CA were informed about the importance of a healthy lifestyle in reducing the risk of cancer. They received a leaflet based on the WCRF/AICR recommendations [14].

Intervention Arm (IA) Program

Dietary caloric-protein restriction

Individualized dietary advice based on the WCRF/AICR recommendations was given to patients randomized in the IA [14]. Advice on a healthy diet was provided to promote the reduction of a patient's total daily calorie intake by up to 600 kcal below their energy requirements. Energy requirements for each patient in the IA were estimated using the revised Harris-Benedict formula [26], which combines basal metabolic rate (BMR) and physical activity level. Caloric restriction involved a dietary intervention aimed at a total protein intake of 0.8g of protein/Kg of body weight, mostly from foods of plant origin.

Patients were required to keep a weekly diet diary that was analyzed using metaDieta 3.5 software (Me.Te.Da. s.r.l., San Benedetto del Tronto, Ascoli Piceno, Italy). Once a month for the first four months and bi-monthly for the rest of the intervention period, patients met with the dietitian on an individual basis for a 45-minute nutritional counseling session. During these visits, patients discussed their diet diary with the dietitian in order to identify the best strategy to follow the restriction program.

Health coaching

After the nutritional counseling session with the dietitian, IA patients met with a trained nurse (coach) for a 15-minute health coaching session. The role of the coach in these sessions was to empower participants to take control of their health, to shift attention from what the professionals want to the patient’s objectives, to help patients to achieve their objectives through feasible steps, and to challenge behaviors that represent an obstacle to positive change.

Cooking classes

Each IA patient participated in a minimum of three 4-hour sessions of culinary practice (cooking classes). Participants were invited to take cooking classes with a relative, such as a wife or a husband. The objective of the practical part of the intervention was to provide patients with skills and knowledge so that they could modify their dietary approach and behaviors to achieve the calorie restriction goal. All sessions and recipes were developed on the basis of the Mediterranean Diet in accordance with the WCRF/AICR recommendations and the Healthy Eating Plate proposed by the Harvard School of Public Health using healthy, mostly local, and seasonal foods [14,27]. Cooking classes were given by a nutritionist and a professional cook. At the end of each cooking class, participants had lunch together, ate the meal they had prepared, and discussed what they had learned with the dietitian.

Physical activity

Following a medical/cardiac evaluation to ascertain their physical ability to exercise, IA patients performed Nordic walking sessions of moderate intensity twice per month during the intervention’s 24-month

duration. A certified expert supervised every Nordic walking session. Patients were counseled with regard to exercising alone or in a group in their usual living environment. IA participants were provided with a pedometer (Omron walking style IV, Omron Healthcare Europe B.V.) to check the number of steps or distance (km) walked each day. Nordic walking sessions were performed in groups of twelve participants. Every session started with 10 minutes of warm-up exercises and continued with moderate intensity walking in a green, pedestrian area. Patients were encouraged to maintain a good level of physical activity during the rest of the week, with 40 minutes of brisk walking 4 days/week. All patients in the IA received reminders of their appointments for nutritional counseling, cooking classes, and Nordic walking sessions through a phone call, e-mail, or text message and were contacted for any changes in the scheduled activities due to unforeseen events.
